# DNA Methylation-Derived Immune Cell Proportions and Cancer Risk in Black Participants

**DOI:** 10.1158/2767-9764.CRC-24-0257

**Published:** 2024-10-17

**Authors:** Christopher S. Semancik, Naisi Zhao, Devin C. Koestler, Eric Boerwinkle, Jan Bressler, Rachel J. Buchsbaum, Karl T. Kelsey, Elizabeth A. Platz, Dominique S. Michaud

**Affiliations:** 1 Department of Public Health and Community Medicine, Tufts University School of Medicine, Tufts University, Boston, Massachusetts.; 2 The University of Kansas Cancer Center, Kansas City, Kansas.; 3 Department of Biostatistics and Data Science, University of Kansas Medical Center, Kansas City, Kansas.; 4 Human Genetics Center, School of Public Health, University of Texas Health Science Center at Houston, Houston, Texas.; 5 Human Genome Sequencing Center, Baylor College of Medicine, Houston, Texas.; 6 Division of Hematology and Oncology, Tufts Medical Center, Boston, Massachusetts.; 7 Department of Epidemiology, Brown University, Providence, Rhode Island.; 8 Department of Pathology and Laboratory Medicine, Brown University, Providence, Rhode Island.; 9 Department of Epidemiology, Johns Hopkins Bloomberg School of Public Health, Baltimore, Maryland.; 10 The Sidney Kimmel Comprehensive Cancer Center at Johns Hopkins, Baltimore, Maryland.

## Abstract

Prior cohort studies assessing cancer risk based on immune cell subtype profiles have predominantly focused on White populations. This limitation obscures vital insights into how cancer risk varies across race. Immune cell subtype proportions were estimated using deconvolution based on leukocyte DNA methylation markers from blood samples collected at baseline on participants without cancer in the Atherosclerosis Risk in Communities Study. During a mean of 17.5 years of follow-up, 668 incident cancers were diagnosed in 2,467 Black participants. Cox proportional hazards regression was used to examine immune cell subtype proportions and overall cancer incidence and site-specific incidence (lung, breast, and prostate cancers). Higher regulatory T-cell proportions were associated with higher lung cancer risk [HR, 1.22; 95% confidence interval (CI), 1.06–1.41 per 1% increase in cell proportion] and a borderline increase in overall cancer risk (*P* = 0.06). Increased memory B-cell proportions were associated with a significantly higher risk of prostate cancer and all cancers (HR, 1.17; 95% CI, 1.04–1.33 and HR, 1.13; 95% CI, 1.05–1.22, per 1% increase in cell proportion, respectively). Other immune cell subtypes did not display statistically significant associations with cancer risk in the main analyses. These results in Black participants align closely with prior findings in largely White populations. Our results add to the growing evidence demonstrating the important role of adaptive immunity in cancer risk.

**Significance:**

This study describes associations between immune cell types and cancer risk in a Black population; elevated regulatory T-cell proportions that were associated with increased overall cancer and lung cancer risk, and elevated memory B-cell proportions that were associated with increased prostate and all cancer risk.

## Introduction

The immune system plays a key role in protecting against cancer (1). Studies using animal models and patients with cancer have shown the immune system’s ability to recognize and eliminate tumor cells through immunosurveillance, which involves both innate and adaptive immune responses (2–4). However, tumor cells can evade immunosurveillance responses by suppressing the immune system (5, 6). Within tumors, higher proportions of cytotoxic T lymphocytes (CD8^+^ cells) have been associated with more favorable cancer outcomes, whereas higher proportions of regulatory T cells (Treg) have been associated with immunosuppression, accelerated cancer development, and decreased survival (7–10). Intratumoral accumulation of Tregs has been consistently associated with greater tumor aggressiveness in patients with various cancer types (11–14).

Despite these insights, the role of peripheral blood immune cell profiles in the precancerous state and their influence on subsequent cancer risk remains unclear. Furthermore, cohort studies cannot systematically use flow cytometry to identify immune cell type profiles in the peripheral blood at regular, consistent intervals because cohorts seldom have whole blood stored under appropriate conditions. To date, few observational studies have examined the relationship between immune cells measured in pre-diagnostic blood and cancer risk (15–23).

Recent advances in high-dimensional arrays enable measurement of DNA methylation at 450,000 to 850,000 CpG oligodeoxynucleotide sites (CpG) throughout the genome, allowing precise estimates of immune cell proportions from frozen blood samples (17–19, 24). Pre-diagnostic blood collection is essential to assess DNA methylation states and immune cell proportions because cancers by themselves may alter these profiles (22). Located throughout the genome, differentially methylated regions can distinctly identify the lineage of differentiated immune cell subtypes (25). These unique differentially methylated CpGs can be used to identify immune cell lineages, and their proportions can be estimated using a statistical method called “deconvolution” (25). The resulting immune cell proportions can be used to assess relationships between immune cell profiles and cancer risk (25). Because DNA methylation analysis can be done using DNA from archived blood, immune profiles can now be assessed using the resources of large epidemiologic studies that have banked specimens.

For this analysis, we used the Atherosclerosis Risk in Communities Study (ARIC) to investigate the risk of lung cancer, breast cancer, prostate cancer, and all cancers pooled together (excluding hematological cancers) in relation to DNA methylation-derived relative proportions of peripheral blood immune cell types in Black participants. We include individual cancers and pooled cancers because our overarching hypothesis is that immune response will impact cancer risk in similar ways, regardless of the tumor type, as systemic inflammation has been linked to a number of different cancers and because many cancers share similar risk factors, such as smoking and obesity. Studying these cancer risks in Black ARIC participants represents an important opportunity to analyze this relationship in an understudied population, given that African ancestry populations are known to have lower average neutrophil counts than European ancestry populations (26, 27).

## Materials and Methods

### Study population

Participants were members of the ARIC study (RRID: SCR_021769), a prospective cohort study of cardiovascular disease risk that enrolled 15,792 people between 1987 and 1989 from four different communities in the United States (Jackson, MS; Washington County, MD; suburban Minneapolis, MN; and Forsyth County, NC; refs. 28, 29). Participants underwent a baseline clinical examination (visit 1; 1987–1989), which included an in-home interview and clinical examination and assessed medical and lifestyle factors (30). Participants returned for follow-up clinical examinations in 1990 to 1992 (visit 2), 1993 to 1995 (visit 3), 1996 to 1998 (visit 4), 2011 to 2013 (visit 5), and so on until visit 10. Blood specimens were banked at each visit, and participants were followed by annual telephone calls until 2011 with semi-annual contact thereafter. The ARIC protocol was conducted in accordance with the U.S. Common Rule, was approved by the institutional review boards at each site, and was agreed to by participants who gave written informed consent.

For this analysis, we included Black participants (initial total of *n* = 2,520) from the Jackson, MS, community (*n* = 2,287) and the Forsyth County, NC, community (*n* = 233) who had previously had methylation profiling performed, fully consented to the cancer and genetic research, and had no cancer history before blood collection. DNA methylation data for the participants with European ancestry were derived for a small initial study by ARIC (938 subjects) at a separate time from the analysis on Black participants. Given the relatively small number of White participants with methylation data, the number of cancer cases during the follow-up period (*n* = 266) was too small to examine with sufficient statistical power.

### Estimation of peripheral blood leukocyte composition

DNA methylation levels were measured on most of the Black participants in the ARIC cohort using archived blood samples collected at visit 2 or visit 3 (31, 32). Genomic DNA was extracted from the peripheral blood leukocyte samples using the Gentra Puregene Blood Kit (QIAGEN), and bisulfite conversion of 1-μg genomic DNA was performed using the EZ-96 DNA Methylation Kit (Deep-Well Format; Zymo Research). The Illumina HumanMethylation450 BeadChip array was used to provide quantitative methylation measurement at 483,525 CpGs in 2,853 Black participants.

As the main exposures of interest, peripheral blood leukocyte subtype proportions were estimated using DNA methylation markers of immune cell lineage for a total of 12 leukocyte subtypes, including myeloid lineage subtypes (neutrophils, eosinophils, basophils, and monocytes) and lymphoid lineage subtypes [B lymphocytes naïve, B lymphocytes memory, T-helper lymphocytes naïve (naïve CD4^+^ cells), T-helper lymphocytes memory (memory CD4^+^ cells), Tregs, cytotoxic T lymphocytes naïve (naïve CD8^+^ cells), cytotoxic T lymphocytes memory (memory CD8^+^ cells), and NK lymphocytes]. This estimation was done using a newly expanded reference-based deconvolution library EPIC IDOL-Ext (33); this library uses the IDOL methodology to deconvolve the proportions of 12 leukocyte subtypes in peripheral blood (34, 35). This EPIC IDOL-Ext library was validated using the gold-standard flow cytometry data and substantiated by including publicly available data from more than 100,000 samples (24, 33, 36–38).

Implausibly low immune cell proportion values were assigned the limit of detection, as described by Bell-Glenn and colleagues (39). Values for the methylation-derived neutrophil-to-lymphocyte ratio (mdNLR) were calculated by dividing neutrophil proportions by lymphocyte proportions and represented as a ratio.

To adjust for batch effects and other unknown technical sources of variation that occurred in the array measurements, we generated surrogate variables using the SVA procedure (ctrlsva R function) as the ComBat method alone was ineffective at adjusting for batch effects (40).

### Prediction smoking score using DNA methylation data

We used the DNA methylation data to derive the pack-years of smoking score which has been previously derived to reflect methylation alterations that are uniquely associated with smoking history and can serve as an additional variable to adjust for smoking effects to further correct for confounding by smoking (41).

### Cancer ascertainment

Based on cancer registry data and follow-up, cancer cases were ascertained for all cancer types, except for non–melanoma skin cancer (Supplementary Table S1). The outcome of interest, cancer incidence of any type, was positive if a participant developed any type of cancer during the follow-up period. The only outcome of interest was first incidences of cancer, not cancer recurrences. For some analyses, the outcome was restricted to the most common types of cancer, including lung cancer, breast cancer, and prostate cancer. In the case of breast cancer, cases in premenopausal women were excluded due to small numbers, meaning that all included cases of breast cancer occurred in postmenopausal women. The premenopausal breast cancer cases, however, were included in all cancers combined. Besides lung, breast, and prostate cancers, other cancers were not analyzed due to the low number of cases associated with the other types of cancers (*n* < 75 cases), as this could resulting in low and reduced statistical power if these cancers were to be analyzed separately. Although premenopausal breast cancer cases were excluded from breast cancer analyses, premenopausal breast cancer cases were included when considering all cancers pooled together.

Incident cancers were ascertained from baseline (either visit 2 or visit 3) until the end of 2015 through linkage with state cancer registries in Minnesota, North Carolina, Maryland, and Mississippi. From baseline through 2015, we ascertained 721 primary cancer cases and 345 cancer deaths in Black participants. These cases occurred during a mean of 17.5 years of follow-up. Furthermore, for those participants who did go on to develop any type of cancer, the mean time from blood draw to cancer diagnosis was 11.4 years. For analyses, all hematological cancers (*n* = 53) were removed, as hematological cancers originate in progenitors that give rise to immune cells and may result in spurious immune profiles. This left 2,467 participants and 668 cancers for analysis.

### Covariate assessment

Risk factors associated with cancer include age, sex, body mass index (BMI), cigarette smoking (self-reported smoking status, self-reported pack-years, and methylation-derived pack-year score; ref. 41), postmenopausal hormone use (reported as current/former/never for postmenopausal women), and alcohol consumption (self-reported drinking status). Data on cigarette smoking and drinking status (current, former, never) and cigarette smoking cumulative dose (pack-years) were collected at each visit during follow-up, and we used the corresponding data from the visit of blood draw. Furthermore, we used the BMI and age data collected at the corresponding visit of blood draw. Postmenopausal hormone use at visit 2 was used and adjusted for in models considering all cancers, breast cancer, or lung cancer, but not prostate cancer (because all cases were male). Race was self-reported by participants. Genetic analyses conducted on the ARIC cohort study participants using ancestral markers have revealed that a median of 15% of self-reported Black participants had European ancestral markers (42). Self-reported education level (basic education: less than completed high school; intermediate education: high school or equivalent; or advanced education: at least some college) and diabetes status were also available for the analyses.

### Measurement of complete blood count and total leukocyte count

In addition to using the deconvolution technique to estimate peripheral blood leukocyte composition for each participant, complete blood count (CBC) was measured in the blood of participants collected at visit 2 (assay was performed within 24 hours of blood draw, after being stored at 4°C). At visit 2, most participants included in this analysis did not have CBC differentials (based on the center of collection) and 30 participants did not have CBC (*n* = 2,437 participants with CBC data).

### Statistical analysis

To estimate the association of methylation-derived immune cell proportions with cancer incidence, we used Cox proportional hazards regression to estimate HRs and 95% confidence intervals (CI) of total cancer, lung cancer, breast cancer, and prostate cancer, adjusting for commonly established risk factors described in the covariate assessment. Frequencies for each type of cancer included in all pooled cancers (*n* = 668) are shown in Supplementary Table S1. Of the lung cancer cases, five were small cell lung cancers, 50 were non–small cell lung cancers, 16 were squamous lung cancers, five were large cell lung cancers, and eight were unclassified. These models indicate change in risk per 1% increase in immune cell proportions, but analyses were also conducted to outline change in risk per standard deviation in immune cell proportions and per difference between the 90th and 10th percentiles (Supplementary Tables S2 and S3). Participants contributed the time at risk from blood draw for profiling at visit 2 (89.1% of participants) or visit 3 (10.9% of participants) until cancer diagnosis of any site, death, or administrative censoring at the end of 2015, whichever came first. To address the possibility that prior unknown preclinical disease may influence immune cell proportions (i.e., reverse causation), we also conducted a time-lag sensitivity analysis removing participants with less than 2 years of follow-up (Supplementary Table S4).

Specifically, the covariates included age (continuous), sex, BMI (continuous), cigarette smoking status, cigarette smoking dose (continuous), postmenopausal hormone use, methylation-derived pack-years (continuous), mdNLR (continuous), drinking status (for breast cancer only), and batch effect (based on surrogate variables; continuous).

Analyses were performed using The R Project for Statistical Computing [v4.0.2; R Core Team 2020 (RRID: SCR_001905)]. Statistical tests were two sided, and a *P* value of less than 0.05 was considered statistically significant. We adjusted the *P* value to account for multiple comparisons using Bonferroni correction.

### Data availability

Supporting data from the ARIC cohort cannot be made openly available; data are available through controlled access. Further information about the data and conditions for access are available at the ARIC website at https://aric.cscc.unc.edu/aric9/.

## Results

Compared to the Black participants in this study, all cancer cases were more likely to be current smokers (with higher pack-years), to be current alcohol drinkers, and women were less likely to be using menopausal hormones (Table 1). Summary data for the different immune cell proportions included in this analysis are provided in Table 2.

**Table 1:** Baseline characteristics for the study population, overall and by cancer types, for Black participants in ARIC

	Incident cancer cases				Overall (*n* = 2,467)
Characteristic	Lung cancer (*n* = 84)	Breast cancer (*n* = 114)	Prostate cancer (*n* = 173)	All cancer (*n* = 668)	
Age					
Mean (SD)	57.7 (6.32)	56.9 (5.66)	56.7 (6.06)	56.9 (5.95)	56.4 (5.80)
Median (min, max)	57.5 (48.0, 68.0)	57.0 (48.0, 70.0)	56.0 (47.0, 68.0)	57.0 (47.0, 70.0)	56.0 (47.0, 71.0)
BMI					
Mean (SD)	28.5 (6.08)	31.5 (6.70)	28.2 (4.73)	30.2 (6.49)	30.1 (6.23)
Median (min, max)	28.4 (18.3, 48.7)	30.5 (17.8, 52.8)	27.7 (19.5, 46.8)	29.3 (16.7, 57.9)	29.3 (14.7, 62.4)
Sex (%)					
Female	37 (44.0%)	114 (100%)	0 (0%)	343 (51.3%)	1,568 (63.6%)
Male	47 (56.0%)	0 (0%)	173 (100%)	325 (48.7%)	899 (36.4%)
Smoking status (%)					
Never	12 (14.3%)	62 (54.4%)	65 (37.6%)	283 (42.4%)	1,226 (49.7%)
Former smoker	20 (23.8%)	22 (19.3%)	59 (34.1%)	171 (25.6%)	626 (25.4%)
Current smoker	52 (61.9%)	30 (26.3%)	49 (28.3%)	214 (32.0%)	615 (24.9%)
Smoking pack-years, never smokers are excluded					
Mean (SD)	36.0 (25.1)	23.4 (17.6)	22.6 (21.1)	25.9 (23.5)	21.9 (22.6)
Median (min, max)	32.4 (0, 141)	21.0 (0, 72.0)	16.6 (0, 114)	21.4 (0, 199)	16.3 (0, 199)
Methylation-derived pack-years					
Mean (SD)	48.0 (50.9)	17.3 (30.9)	25.8 (40.9)	24.9 (39.3)	20.4 (35.5)
Median (min, max)	31.5 (0, 196)	0 (0, 193)	0 (0, 202)	0 (0, 202)	0 (0, 302)
Menopausal hormone therapy use, women only (%)					
Never	25 (67.6%)	71 (62.2%)	0 (0%)	214 (62.4%)	954 (60.8%)
Former user	1 (2.7%)	0 (0%)	0 (0%)	8 (2.3%)	44 (2.8%)
Current user	5 (13.5%)	24 (21.1%)	0 (0%)	71 (20.7%)	291 (18.6%)
Unknown	6 (16.2%)	19 (16.7%)	0 (0%)	50 (14.6%)	279 (17.8%)
Education level (%)					
Basic education or no education	42 (50.0%)	47 (41.2%)	62 (35.8%)	264 (39.5%)	971 (39.4%)
Intermediate education	25 (29.8%)	35 (30.7%)	47 (27.2%)	196 (29.3%)	704 (28.5%)
Advanced education	17 (20.2%)	32 (28.1%)	64 (37.0%)	207 (31.1%)	784 (31.8%)
Missing	0 (0%)	0 (0%)	0 (0%)	1 (0.1%)	8 (0.3%)
Drinking status (%)					
Never	19 (22.6%)	49 (43.0%)	30 (17.3%)	210 (31.4%)	867 (35.1%)
Former drinker	27 (32.1%)	39 (34.2%)	52 (30.1%)	207 (31.0%)	781 (31.7%)
Current drinker	38 (45.2%)	26 (22.8%)	91 (52.6%)	251 (37.6%)	819 (33.2%)
Treg proportion					
Mean (SD)	2.82 (2.47)	2.49 (1.72)	2.06 (1.70)	2.28 (1.88)	2.14 (1.76)
Median (min, max)	2.05 (0.795, 12.4)	2.29 (0.795, 8.16)	1.42 (0.795, 13.7)	1.77 (0.795, 14.2)	1.57 (0.795, 14.9)
CD8^+^ naïve cell proportion					
Mean (SD)	1.62 (1.49)	2.07 (1.80)	1.65 (1.44)	1.81 (1.55)	1.95 (1.68)
Median (min, max)	0.793 (0.793, 9.15)	0.793 (0.793, 7.45)	0.886 (0.793, 8.54)	0.903 (0.793, 9.15)	1.04 (0.793, 16.1)
B-cell memory proportion					
Mean (SD)	0.682 (0.902)	0.620 (0.630)	0.909 (1.36)	0.793 (1.04)	0.690 (0.910)
Median (min, max)	0.384 (0.384, 16.0)	0.384 (0.384, 3.85)	0.384 (0.384, 9.92)	0.384 (0.384, 9.92)	0.384 (0.384, 16.0)
mdNLR					
Mean (SD)	1.92 (3.92)	1.98 (4.39)	2.51 (5.06)	2.24 (4.14)	2.21 (4.02)
Median (min, max)	1.32 (0.176, 46.3)	1.23 (0.263, 46.3)	1.38 (0.304, 46.3)	1.41 (0.263, 46.3)	1.32 (0.176, 46.3)

**Table 2:** Immune cell profiles (mean, SD, median, and range) overall and by cancer status

Cell count or subtype	No cancer (*n* = 1799)	All cancer (*n* = 668)	Overall (*n* = 2,467)
CD4^+^ (%)			
Mean (SD)	13.9 (7.79)	13.3 (7.63)	13.7 (7.75)
Median (min, max)	13.3 (0.962, 46.8)	12.7 (0.962, 41.2)	13.1 (0.962, 46.8)
Memory CD4^+^ (%)			
Mean (SD)	8.24 (5.94)	7.99 (5.90)	8.17 (5.93)
Median (min, max)	7.36 (1.06, 34.1)	7.30 (1.06, 34.1)	7.32 (1.06, 34.1)
Naïve CD4^+^ (%)			
Mean (SD)	5.89 (4.51)	5.54 (4.41)	5.79 (4.48)
Median (min, max)	5.02 (0.863, 36.5)	4.62 (0.863, 25.3)	4.97 (0.863, 36.5)
CD8^+^ (%)			
Mean (SD)	12.9 (6.67)	12.7 (6.71)	12.8 (6.68)
Median (min, max)	12.2 (0.776, 50.7)	11.9 (0.776, 44.7)	12.1 (0.776, 50.7)
Memory CD8^+^ (%)			
Mean (SD)	11.2 (7.04)	11.3 (7.17)	11.2 (7.07)
Median (min, max)	10.2 (0.758, 50.7)	9.82 (0.758, 44.7)	10.1 (0.758, 50.7)
Naïve CD8^+^ (%)			
Mean (SD)	2.00 (1.73)	1.81 (1.55)	1.95 (1.68)
Median (min, max)	1.10 (0.793, 16.1)	0.903 (0.793, 9.15)	1.04 (0.793, 16.1)
Treg (%)			
Mean (SD)	2.09 (1.71)	2.28 (1.88)	2.14 (1.76)
Median (min, max)	1.51 (0.795, 14.9)	1.77 (0.795, 14.2)	1.57 (0.795, 14.9)
B cell (%)			
Mean (SD)	5.32 (3.26)	5.21 (3.09)	5.30 (3.21)
Median (min, max)	4.80 (0.423, 26.1)	4.82 (0.423, 19.3)	4.80 (0.423, 26.1)
Memory B cell (%)			
Mean (SD)	0.65 (0.85)	0.79 (1.04)	0.69 (0.91)
Median (min, max)	0.38 (0.384, 16.0)	0.38 (0.384, 9.9)	0.38 (0.384, 16.0)
Naïve B cell (%)			
Mean (SD)	4.96 (3.19)	4.70 (3.02)	4.89 (3.15)
Median (min, max)	4.53 (0.461, 26.1)	4.24 (0.461, 19.3)	4.41 (0.461, 26.1)
Neutrophil (%)			
Mean (SD)	49.9 (14.3)	50.8 (14.3)	50.1 (14.3)
Median (min, max)	48.4 (13.1, 100)	49.9 (18.1, 100)	49.0 (13.1, 100)
Monocyte (%)			
Mean (SD)	10.2 (2.81)	10.1 (2.85)	10.2 (2.82)
Median (min, max)	10.1 (0.359, 23.8)	9.98 (0.359, 22.5)	10.1 (0.359, 23.8)
NK cell (%)			
Mean (SD)	4.62 (2.55)	4.53 (2.55)	4.60 (2.55)
Median (min, max)	4.20 (0.488, 20.7)	4.06 (0.488, 19.1)	4.19 (0.488, 20.7)
White blood cell count (×1,000 mm^3^)			
Mean (SD)	5.59 (1.84)	5.72 (1.78)	5.62 (1.82)
Median (min, max)	5.30 (2.00, 18.7)	5.40 (2.40, 16.9)	5.30 (2.00, 18.7)
Missing	21 (1.2%)	9 (1.3%)	30 (1.2%)

Many of the associations observed for the 12 immune cell subtypes and ratios examined were not statistically significantly associated with overall cancer risk (Table 3). However, consistent patterns were noted for three immune cell subtypes: Tregs, naïve CD8^+^ cells, and memory B cells, in addition to overall white blood cell count.

**Table 3:** HRs for immune cell proportions and cancer risk in black participants in ARIC. HR^a^ (95% CI) per 1% increase in methylation-derived immune cell proportion, one unit increase in ratios or per 1000 white blood cell count (mm^3^)

Methylation-derived immune cell type or measure	All cancer^b^ (668 cases)	Lung cancer (84 cases)	Postmenopausal breast cancer^c^ (114 cases)	Prostate cancer^d^ (173 cases)
CD4^+^	0.99 (0.98, 1.01)	1.00 (0.96, 1.03)	1.00 (0.97, 1.02)	1.00 (0.98, 1.03)
Memory CD4^+^	0.99 (0.98, 1.01)	0.99 (0.95, 1.04)	1.01 (0.98, 1.05)	1.00 (0.97, 1.03)
Naïve CD4^+^	1.00 (0.98, 1.02)	1.00 (0.95, 1.06)	0.97 (0.93, 1.01)	1.01 (0.97, 1.06)
Naïve-to-memory CD4^+^ ratio	1.02 (0.98, 1.05)	0.96 (0.85, 1.09)	1.00 (0.93, 1.08)	0.98 (0.90, 1.06)
CD8^+^	1.00 (0.99, 1.01)	1.02 (0.99, 1.06)	1.02 (0.98, 1.05)	1.01 (0.99, 1.03)
Memory CD8^+^	1.00 (0.99, 1.02)	1.03 (1.00, 1.06)	1.02 (0.99, 1.05)	1.01 (0.99, 1.03)
Naïve CD8^+^	0.96 (0.91, 1.01)	0.85 (0.71, 1.01)	0.96 (0.86, 1.07)	1.00 (0.89, 1.12)
Naïve-to-memory CD8^+^ ratio	0.98 (0.87, 1.09)	0.87 (0.59, 1.29)	0.96 (0.75, 1.24)	0.84 (0.62, 1.13)
CD4^+^-to-CD8^+^ ratio	0.97 (0.91, 1.04)	0.98 (0.84, 1.15)	0.89 (0.75, 1.07)	0.94 (0.82, 1.07)
Treg	1.06 (1.00, 1.12)	**1.22 (1.06, 1.41)**	1.04 (0.92, 1.18)	1.08 (0.96, 1.22)
B cell	0.99 (0.97, 1.02)	0.98 (0.91, 1.06)	1.04 (0.98, 1.10)	0.97 (0.92, 1.03)
Memory B cell	**1.13 (1.05, 1.22)**	1.20 (0.99, 1.46)	0.95 (0.74, 1.24)	**1.17 (1.04, 1.33)**
Naïve B cell	0.98 (0.95, 1.01)	0.96 (0.88, 1.04)	1.04 (0.98, 1.10)	0.94 (0.89, 1.00)
Naïve-to-memory B-cell ratio	0.99 (0.98, 1.00)	1.06 (0.94, 1.00)	1.01 (0.99, 1.04)	0.98 (0.96, 1.00)
NLR	1.00 (0.98, 1.02)	0.95 (0.87, 1.03)	0.99 (0.94, 1.04)	1.01 (0.98, 1.04)
Lymphocyte-to-monocyte ratio	1.00 (1.00, 1.00)	1.00 (1.00, 1.00)	1.00 (1.00, 1.00)	1.03 (0.95, 1.12)
White blood cell count (increment per 1000 mm^3^)	1.03 (0.98, 1.08) (*n* = 659)	**1.19 (1.06, 1.33)** (*n* = 83)	1.05 (0.94, 1.18) (*n* = 112)	0.99 (0.91, 1.08) (*n* = 171)

Abbreviation: NLR, neutrophil-to-lymphocyte ratio.

Bold value indicates *P* < 0.05.

a Multivariable models were adjusted for age, sex, BMI, self-reported smoking status, self-reported smoking pack-years, postmenopausal hormone use (creating an “NA” category for males), methylation-derived smoking pack-years, mdNLR (in all models except mdNLR and lymphocyte-to-monocyte ratio), and batch effect.

b Excluding hematologic cancers.

c The breast cancer model also adjusted for self-reported drinking status and not adjusted for sex.

d The prostate cancer model not adjusted for sex or postmenopausal hormone use (men only).

**Figure 1:**
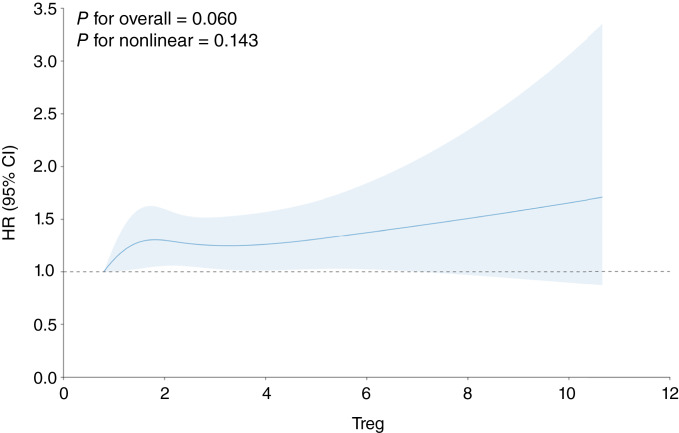
All cancer spline plot. The spline plot assesses the change in the HRs at various proportions of Treg cells in all cancers.

**Figure 2:**
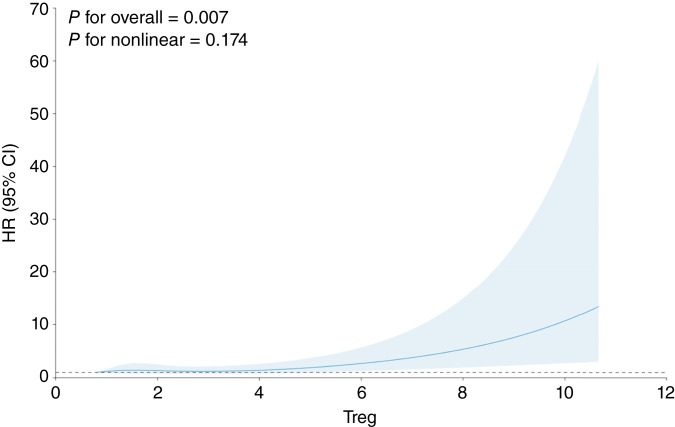
Lung cancer spline plot. The spline plot assesses the change in the HRs at various proportions of Treg cells in lung cancer.

Assessing Treg proportions, each 1% increase was associated with a 6% increased risk of all cancers (HR, 1.06; 95% CI, 1.00–1.11; Fig. 1; Table 3). For lung cancer, a 1% increase of Treg proportion was associated with a 22% increased risk of lung cancer (HR, 1.22; 95% CI, 1.06–1.41; Fig. 2; Table 3). Treg proportions were associated with increased risk of breast cancer and prostate cancer but were not statistically significant (Table 3; Supplementary Figs. S1 and S2). Treg associations for all cancers and lung cancer did not vary substantially when stratified by age (of 55 years or younger vs. more than 55 years) or by sex (Supplementary Tables S5 and S6). Mutually adjusting for measured total leukocyte count and methylation-derived Treg proportions did not attenuate findings for either measure.

Although findings were only borderline statistically significant for naïve CD8^+^ cell proportions, inverse associations were observed for total cancer and lung cancer. A 1% increased naïve CD8^+^ was associated with a 4% decreased risk of all cancers (HR, 0.96; 95% CI, 0.91–1.01; Table 3). Each 1% increase in naïve CD8^+^ cell proportion was associated with a 15% decreased risk of lung cancer (HR, 0.85; 95% CI, 0.71–1.01; Table 3). Naïve CD8^+^ cell proportion was not associated with breast cancer (in the continuous analysis) or prostate cancer risk (Table 3).

Assessing memory B-cell proportions, each 1% increase was associated with a 13% increased risk of all cancers (HR, 1.13; 95% CI, 1.05–1.22), a 17% increased risk of prostate cancer (HR, 1.17; 95% CI, 1.04–1.33), and a 20% increased risk of lung cancer (HR, 1.20; 95% CI, 0.99–1.46; Table 3). Because prostate cancer affects some men minimally and others quite aggressively, an analysis was conducted for prostate cancer cases without a lethal phenotype (i.e., cases that were not metastatic at diagnosis or during follow-up and were not fatal cases; *n* = 151); a similar association was observed for memory B cells (HR, 1.19; 95% CI, 1.05–1.34). By contrast, no association was noted for memory B cells and breast cancer risk. The associations for memory B-cells were statistically significant for all cancers in men (HR, 1.16; 95% CI, 1.05–1.28) but not women, and in lung cancer for men (HR, 1.34; 95% CI, 1.04–1.71) but not women (Supplementary Tables S5 and S6).

In order to assess the potential of chance findings due to multiple comparisons, we applied the Bonferroni correction (16 tests; adjusted significance level of *P* = 0.05/16 = 0.003). When using this significance threshold, the lung cancer Treg result and prostate cancer memory B cell result are no longer statistically significant.

Although the associations for Tregs, naïve CD8^+^ cells, and memory B cells for all cancers and lung cancer followed a linear dose–response, spline plots showed that Treg and naïve CD8^+^ cell associations for breast and prostate cancers follow a nonlinear pattern (Supplementary Figs. S1 and S2). Therefore, we conducted quantile-based analyses for all cancers and individual cancers to address nuance in relationships modeled in previously discussed continuous models. The percentiles used to create the quantiles for each immune cell subtype are provided in Supplementary Tables S7 and S8. In the quartile analysis for all cancers, a 31% elevated risk of all cancers was observed for the highest quartile of Treg proportion, relative to the lowest quartile (HR, 1.31; 95% CI, 1.04–1.65; Supplementary Table S9). Furthermore, a 47% increased risk of all cancers was observed for the highest quartile compared with the lowest quartile of memory B-cell proportion (HR, 1.47; 95% CI, 1.13–1.91; Supplementary Table S9).

Using tertiles for the lung cancer analysis (due to smaller number of cases), there was a dose–response but not a statistically significant relationship for Treg and memory B-cell proportion, whereas for naïve CD8^+^ cell proportion, a statistically significant relationship was observed for the highest tertile when compared with the lowest tertile (HR, 0.47; 95% CI, 0.25–0.87; Supplementary Table S10). For white blood cell count, a 167% elevated risk of lung cancer was observed for the highest tertile when compared with the lowest tertile of white blood cell count (HR, 2.67; 95% CI, 1.43–4.97; Supplementary Table S10). For breast cancer, relationships were generally less dose–response oriented, but statistically significant inverse associations were noted for naïve CD8^+^ in both tertiles (Supplementary Table S11). Only white blood cell count followed a somewhat dose–response–oriented relationship for breast cancer. For prostate cancer, associations were similarly nonlinear (Supplementary Table S12). Furthermore, when excluding individuals with white blood cell counts outside of the reference range for Black men and women (women: 3.4−11.0 × 10^9^/L, men: 3.1−9.9 × 10^9^/L), similar patterns were seen (Supplementary Table S13; ref. 43).

## Discussion

Our study uniquely highlights that in a Black population, higher methylation-derived peripheral blood Treg proportion was associated with elevated risk of lung cancer, even when adjusting for smoking status and pack-years. Furthermore, for Treg proportion, a significant association was observed for overall cancer in the quartile analysis. Additionally, increased memory B-cell proportion was associated with elevated risk of all cancers, prostate cancer, and lung cancer. Conversely, naïve CD8^+^ cell proportion was associated with a suggestive decrease in risk of lung cancer and all cancers, an association that was statistically significant in a sensitivity analysis removing WBC out of the normal range (Supplemental Table S13). A positive association between total white blood cell count (directly measured) and lung cancer risk was also observed, which was independent of relationships measured in methylation-derived immune cell subtypes. Our findings extend prior findings that examined immune cell profiles and risks of major cancer types to a new population, using a cutting-edge algorithm for predicting immune cell subtype proportions.

Our work is among the first to investigate these associations in a Black cohort. Nonetheless, our findings merit comparison to other existing studies. Several studies have addressed the association between total white blood cell count and cancer risk. Studies conducted in the Women’s Health Initiative and UK Biobank cohorts have reported that elevated white blood cell counts are associated with statistically significant increases in the risk of invasive breast cancer in postmenopausal women, and of endometrial cancer and lung cancer in a general cohort of individuals of ages between 40 and 69 years (15, 16). Fewer studies have examined immune cell subtypes. Generally, higher proportions of Tregs relative to total leukocytes or other immune cells have been associated with higher risks of lung, colorectal, breast, and pancreatic cancers (17–19). On the other hand, higher relative proportions of CD8^+^ cells have been inversely associated with the risk of lung cancer, breast cancer, and pancreatic cancer (17, 19). Using a different cohort (the CLUE study), we previously detected a statistically significant increase in the risk of non–small cell lung cancer for an increase of one standard deviation in mdNLR (20). Additionally, an article in 2020 by Kresovich and colleagues (21) reported that increased B-cell proportions are associated with higher breast cancer risk and that increased monocyte proportions are associated with lower breast cancer risk among premenopausal women. By contrast, no statistically significant associations were observed between four measures of immune cell proportions (mdNLR, total CD4^+^/CD8^+^ cells, B cells/lymphocytes, and T cells/lymphocytes) and the risk of pancreatic cancer in a separate study (22). To our knowledge, no study has examined associations between immune cell subtypes and prostate cancer risk.

In addition to observational studies, investigations into cancer cells, hosts, and microenvironments have postulated mechanisms through which cancerous cells are eliminated efficiently by CD8^+^ cells, although Tregs weaken cellular immune response by impeding the activation of effector T cells, preventing cancer cells from being destroyed and promoting tumor growth (2, 3, 44). This induces cellular and molecular networks, which induce an immunosuppressive environment that favors tumor growth (45–47). Increased ratios of Tregs to CD8^+^ cells have been shown to be indicators of this immune evasion and tumor growth within the tumor microenvironment (48–50). It is still largely unknown how Tregs and B cells may interplay to initiate or accelerate tumorigenesis. On the other hand, naïve CD8^+^ cells are preferential immune cell types for targeting and preventing carcinogenesis (51). During carcinogenesis, CD8^+^ cells encounter dysfunction and exhaustion due to immune-related tolerance and immunosuppression within the tumor microenvironment, which favors adaptive immune resistance (51). Upon their activation, CD8^+^ cells infiltrate to the core of the invading site of tumors and kill cancer cells (51). By killing malignant cells upon recognition of specific antigenic peptides by T-cell receptors, CD8^+^ cells play a central protective role in cancer immunity, unlike Tregs (52).

There are many key strengths to this analysis. First, the ARIC is a prospective cohort with a large number of Black participants (28). Prior studies using cohorts such as EPIC, Women’s Health Initiative, UK Biobank, the Sister Study, and CLUE II have focused on White populations, due to the composition of those populations (17–19, 23). To our knowledge, no existing study has evaluated the association between methylation-derived immune cell subtypes and cancer risk in a Black study population, extending prior findings into a historically under-researched population. Furthermore, many prior studies have focused only on major immune cell types and have not studied immune cell subtypes such as Tregs, naïve CD8^+^ cells, and memory B cells. This analysis uses the innovative deconvolution algorithm to predict proportions of 12 distinct immune cell subtypes (22, 44, 51). Unlike previously used methods, deconvolution allows for immune cell proportions to be estimated and measured from archived blood (peripheral blood leukocytes), allowing for a prospective study design that examines the role of systemic immune response in cancer risk (19, 25).

One key limitation of this study is the relatively small number of individual cancer cases, such as lung cancer (*n* = 84 cases). Despite this, statistically significant associations observed indicate robust findings. However, larger sample sizes are necessary for a more detailed analysis of specific cancer types, such as colorectal cancer (*n* = 67 cases). We cannot rule out measurement error (including batch-to-batch variation in array data), residual confounding, and reverse causation (including undetected cancer incidence increasing immune cell proportions during short follow-up periods; refs. 19, 25). Although cohort studies inherently carry risks of such biases, we have accounted for them through comprehensive multivariable models, sensitivity analyses, time-lag analyses, adjustment for batch effect by accounting for heterogeneity of measurements at CpGs, and a biological measure of smoking pack-years. Taken together, accounting for these biases allows us to conclude that undetected, developing, or incident cancers likely do not drive increased or decreased cell proportions and that residual confounding or measurement error minimally contribute to the observed results. Using the Bonferroni correction for multiple comparisons (16 tests; adjusted significance level of *P* = 0.05/16 = 0.003), some findings are no longer statistically significant (Tregs in lung cancer and memory B cells in prostate cancer), so these results should be interpreted with caution as they could represent chance findings. However, our findings are largely consistent with prior studies, so results are likely not due to chance alone. Another limitation of this study is that the number of White ARIC participants with DNA methylation was too small to examine with sufficient statistical power. Therefore, no direct comparison was made between immune cell profiles and cancer risks of White and Black ARIC participants in this current analysis. Finally, because only postmenopausal women were included in breast cancer analysis, we could not directly replicate a prior B-cell finding in premenopausal women (21).

In summary, this study shows that in a Black population, higher methylation-derived proportion of Tregs is associated with increased risk of overall cancer and lung cancer, and a higher memory B-cell proportion is associated with increased risk of all cancers, prostate cancer, and lung cancer. By contrast, higher naïve CD8^+^ cell proportion is associated with decreased risk of lung cancer, breast cancer, and all cancers. Our study confirms the complex interplay between various immune cell types and cancer risk in a Black population, which has been observed in non–Black populations (15–23). These findings contribute significantly to our understanding of the role of immunology in cancer risk; future studies should confirm some of the new associations identified in this cohort.

## Supplementary Material

Tables 1-13, Figures S1-S2
