# Supplementary material for: DNA Methylation-Derived Immune Cell Proportions and Cancer Risk in Black Participants
Source: Cancer Res Commun. 2024 Oct 17;4(10):2714–23. doi: 10.1158/2767-9764.CRC-24-0257 (PMC11484294; doi:10.1158/2767-9764.CRC-24-0257)
Supplement: Supplementary file 1 — Tables 1-13, Figures S1-S2 [file crc-24-0257_supplementary_data_suppst1_st13sf1-sf2.docx]

**8/22/2024**

**Supplementary Materials for Article: DNA Methylation-Derived Immune Cell Proportions and Cancer Risk in Black Participants**

Authors: Christopher S. Semancik, M.P.H., B.S.^1^, Naisi Zhao, M.S., Dr.PH^1^, Devin C. Koestler, Ph.D.^2,3^, Eric Boerwinkle, Ph.D.^4,5^, Jan Bressler, Ph.D.^4^, Rachel J. Buchsbaum, M.D.^6^, Karl T. Kelsey, M.D., M.O.H.^7,8^, Elizabeth A. Platz, Sc.D, M.P.H.^9,10^, Dominique S. Michaud, Sc.D.^1^

**Author Affiliations:**

^1^Department of Public Health & Community Medicine, Tufts University School of Medicine, Tufts University, Boston, MA, USA

^2^The University of Kansas Cancer Center, Kansas City, KS, USA

^3^Department of Biostatistics & Data Science, University of Kansas Medical Center, Kansas City, KS, USA

^4^Human Genetics Center, School of Public Health, University of Texas Health Science Center at Houston, Houston, TX, USA

^5^Human Genome Sequencing Center, Baylor College of Medicine, Houston, TX, USA

^6^Division of Hematology/Oncology, Tufts Medical Center, Boston, MA, USA

^7^Department of Epidemiology, Brown University, Providence, RI, USA

^8^Department of Pathology and Laboratory Medicine, Brown University, Providence, RI, USA

^9^Department of Epidemiology, Johns Hopkins Bloomberg School of Public Health, Baltimore, MD, USA

^10^The Sidney Kimmel Comprehensive Cancer Center at Johns Hopkins, Baltimore, MD, USA

**Corresponding Author:**

Dominique S. Michaud, ScD

Tufts University School of Medicine

136 Harrison Avenue

Boston, MA 02111

Tel: ​617-636-0482

Email: [Dominique.Michaud@tufts.edu](mailto:Dominique.Michaud@tufts.edu)

**Keywords:** DNA methylation, immune cell profiles, deconvolution, immunology, epidemiology

Supplementary Table 1: Cancer Types and Frequencies Included in All Cancers:

| Cancer Type | Cancer Frequency |
| --- | --- |
| Head/Neck Cancer | 7 |
| Colon Cancer | 67 |
| Rectal Cancer | 11 |
| Liver Cancer | 7 |
| Pancreatic Cancer | 31 |
| Stomach Cancer | 16 |
| Other Digestive Cancer | 18 |
| **Lung Cancer** | **84** |
| Other Respiratory Cancer | 7 |
| **Breast Cancer** | **115** |
| Cervical Cancer | 6 |
| Endometrial Cancer | 16 |
| Ovarian/Other Uterine Adnexa Cancer | 7 |
| **Prostate Cancer** | **173** |
| Bladder Cancer | 17 |
| Kidney/Other Urinary Cancer | 32 |
| Brain/Central Nervous System Cancer | 11 |
| Thyroid Cancer | 5 |
| Unspecified Cancer | 14 |
| Cancer of Other Specified Site | 24 |

Note: Bolded cancers were analyzed individually.

Supplementary Table 2: HRs for All Cancers^a^ for 1 SD Increment:

HR^b^ (95% CI) per 1 standard deviation increase in methylation-derived immune cell proportion

| Methylation-Derived Immune Cell Type or Measure | All Cancer  (668 cases) |
| --- | --- |
| CD4+ | 0.96 (0.87, 1.05) |
| Memory CD4+ | 0.95 (0.87, 1.04) |
| Naïve CD4+ | 1.00 (0.92, 1.09) |
| Naïve-to-memory CD4+ ratio | 1.04 (0.96, 1.13) |
| CD8+ | 1.01 (0.93, 1.10) |
| Memory CD8+ | 1.03 (0.95, 1.12) |
| Naïve CD8+ | 0.93 (0.85, 1.01) |
| Naïve-to-memory CD8+ ratio | 0.98 (0.91, 1.07) |
| CD4+-to-CD8+ ratio | 0.97 (0.89, 1.04) |
| Treg | 1.10 (1.00, 1.21) |
| B cell | 0.98 (0.90, 1.07) |
| Memory B cell | **1.12 (1.05, 1.19)** |
| Naïve B cell | 0.94 (0.86, 1.02) |
| Naïve-to-memory B cell ratio | 0.92 (0.85, 1.01) |
| NLR | 1.00 (0.93, 1.08) |
| Lymphocyte to Monocyte ratio | 0.92 (0.47, 1.81) |
| White blood cell count | 1.05 (0.97, 1.14)  (n = 659) |

^a^Excluding hematological cancers. This table shows hazard ratios for all cancer incidence, interpreted as increase in hazard per increase of one standard deviation, rather than per one-percent increase in cell proportion, like in the rest of the tables. However, for most methylation-derived immune cell subtypes, standard deviations are relatively close to 1%, as represented in all other models.

^b^This multivariable model has been adjusted for age, sex, BMI, self-reported smoking status, self-reported smoking pack-years, methylation-derived smoking pack-years, postmenopausal hormone use, mdNLR, and batch effect.

Supplementary Table 3: HRs ^a^ for Difference Between 10^th^ Percentile and 90^th^ Percentile:

| Methylation-Derived Immune Cell Type or Measure | All Cancer ^b^  (668 cases) | Lung Cancer  (84 cases) | Postmenopausal Breast Cancer ^c^  (114 cases) | Prostate Cancer ^d^  (173 cases) |
| --- | --- | --- | --- | --- |
| CD4+ | 0.89 (0.70, 1.13) | 0.91 (0.44, 1.91) | 0.92 (0.54, 1.59) | 1.11 (0.67, 1.82) |
| Memory CD4+ | 0.88 (0.70, 1.10) | 0.91 (0.47, 1.79) | 1.24 (0.74, 2.07) | 1.02 (0.64, 1.60) |
| Naïve CD4+ | 1.00 (0.81, 1.23) | 0.99 (0.54, 1.82) | 0.71 (0.44, 1.17) | 1.15 (0.72, 1.82) |
| Naïve-to-memory CD4+ ratio | 1.07 (0.94, 1.23) | 0.86 (0.52, 1.43) | 1.01 (0.76, 1.35) | 0.92 (0.66,1.28) |
| CD8+ | 1.02 (0.83, 1.25) | 1.50 (0.85, 2.64) | 1.28 (0.77, 2.11) | 1.21 (0.84, 1.73) |
| Memory CD8+ | 1.07 (0.88, 1.31) | 1.68 (0.98, 2.90) | 1.36 (0.84, 2.22) | 1.17 (0.82, 1.68) |
| Naïve CD8+ | 0.86 (0.71, 1.03) | 0.56 (0.31, 1.02) | 0.87 (0.59, 1.28) | 1.01 (0.68, 1.49) |
| Naïve-to-memory CD8+ ratio | 0.98 (0.88, 1.09) | 0.88 (0.60, 1.27) | 0.96 (0.76, 1.23) | 0.84 (0.63, 1.12) |
| CD4+-to-CD8+ ratio | 0.94 (0.82, 1.08) | 0.96 (0.68, 1.36) | 0.78 (0.52 (1.17) | 0.87 (0.65, 1.17) |
| Treg | 1.20 (1.00, 1.45) | **1.95 (1.20, 3.18)** | 1.15 (0.76, 1.75) | 1.31 (0.87, 1.97) |
| B cell | 0.95 (0.77, 1.17) | 0.87 (0.47, 1.62) | 1.33 (0.85, 2.08) | 0.80 (0.51, 1.25) |
| Memory B cell | **1.13 (1.05, 1.22)** | 1.21 (0.99, 1.48) | 0.95 (0.73, 1.25) | **1.18 (1.04, 1.34)** |
| Naïve B cell | 0.85 (0.69, 1.06) | 0.70 (0.37, 1.34) | 1.36 (0.88, 2.10) | 0.63 (0.39, 1.02) |
| Naïve-to-memory B cell ratio | 0.83 (0.67, 1.02) | 0.52 (0.27, 1.03) | 1.35 (0.87, 2.09) | 0.67 (0.41, 1.08) |
| NLR | 1.00 (0.95, 1.06) | 0.85 (0.65, 1.10) | 0.97 (0.83, 1.13) | 1.03 (0.93, 1.13) |
| Lymphocyte to Monocyte ratio | 1.00 (1.00, 1.00) | 1.00 (1.00, 1.00) | 1.00 (1.00, 1.00) | 1.13 (0.81, 1.56) |
| White blood cell count | 1.13 (0.93, 1.37)  (n = 659) | **2.09 (1.30, 3.35)**  (n = 83) | 1.26 (0.77, 2.06)  (n = 112) | 0.95 (0.65, 1.39)  (n = 171) |

^a^ This multivariable model has been adjusted for age, sex, BMI, self-reported smoking status, self-reported smoking pack-years, methylation-derived smoking pack-years, postmenopausal hormone use, mdNLR, and batch effect.

^b^ Excluding hematological cancers. This table shows hazard ratios for all cancer incidence, interpreted as increase in hazard per difference between 10^th^ and 90^th^ percentile, rather than per one-percent increase in cell proportion, like in the rest of the tables.

^c^ The breast cancer model also adjusted for self-reported drinking status and did not adjust for sex.

^d^ The prostate cancer model did not adjust for sex or postmenopausal hormone use (men only).

Supplementary Table 4: HRs for Time-Lag Analysis:

HR ^a^ (95% CI) per 1 percent increase in methylation-derived immune cell proportion or 1 unit increase in ratios or white blood cell count

| Methylation-Derived Immune Cell Type or Measure | All Cancer ^b^  (625 cases) | Lung Cancer  (79 cases) | Postmenopausal Breast Cancer ^c^  (106 cases) | Prostate Cancer ^d^  (163 cases) |
| --- | --- | --- | --- | --- |
| CD4+ | 1.00 (0.98, 1.01) | 1.00 (0.96, 1.04) | 1.00 (0.97, 1.03) | 1.00 (0.98, 1.03) |
| Memory CD4+ | 0.99 (0.98, 1.01) | 1.00 (0.95, 1.05) | 1.02 (0.98, 1.05) | 1.00 (0.97, 1.03) |
| Naïve CD4+ | 1.00 (0.98, 1.02) | 1.00 (0.95, 1.06) | 0.97 (0.93, 1.02) | 1.00 (0.96, 1.05) |
| Naïve-to-memory CD4+ ratio | 1.02 (0.98, 1.05) | 0.96 (0.84, 1.09) | 0.99 (0.91, 1.08) | 0.96 (0.88, 1.05) |
| CD8+ | 1.00 (0.99, 1.01) | 1.03 (1.00, 1.07) | 1.01 (0.98, 1.04) | 1.01 (0.99, 1.03) |
| Memory CD8+ | 1.00 (0.99, 1.02) | 1.04 (1.00, 1.07) | 1.02 (0.99, 1.05) | 1.01 (0.99, 1.03) |
| Naïve CD8+ | 0.95 (0.90, 1.01) | 0.86 (0.72, 1.02) | 0.95 (0.84, 1.07) | 1.00 (0.89, 1.12) |
| Naïve-to-memory CD8+ ratio | 0.97 (0.87, 1.09) | 0.89 (0.60, 1.32) | 0.93 (0.70, 1.23) | 0.83 (0.61, 1.14) |
| CD4+-to-CD8+ ratio | 0.98 (0.92, 1.05) | 0.99 (0.85, 1.15) | 0.90 (0.75, 1.09) | 0.94 (0.82, 1.08) |
| Treg | 1.06 (1.00, 1.12) | **1.25 (1.08, 1.44)** | 1.04 (0.91, 1.18) | 1.12 (0.98 (1.26) |
| B cell | 1.00 (0.97, 1.03) | 0.99 (0.91, 1.07) | 1.04 (0.98, 1.11) | 0.98 (0.93, 1.04) |
| Memory B cell | **1.14 (1.06, 1.23)** | 1.21 (1.00, 1.48) | 0.95 (0.72, 1.24) | **1.19 (1.05, 1.34)** |
| Naïve B cell | 0.98 (0.96, 1.01) | 0.96 (0.88, 1.05) | 1.05 (0.99, 1.11) | 0.95 (0.90, 1.01) |
| Naïve-to-memory B cell ratio | 0.99 (0.98, 1.00) | 0.97 (0.94, 1.01) | 1.02 (0.99, 1.04) | 0.98 (0.96, 1.01) |
| NLR | 1.00 (0.98, 1.02) | 0.94 (0.85, 1.03) | 0.99 (0.94, 1.04) | 1.03 (0.99, 1.06) |
| Lymphocyte to Monocyte ratio | 1.00 (1.00, 1.00) | 1.00 (1.00, 1.00) | 1.00 (1.00, 1.00) | 1.00 (0.88, 1.13) |
| White blood cell count | 1.02 (0.98, 1.07)  (n = 616) | **1.20 (1.07, 1.34)**  (n = 78) | 1.04 (0.92, 1.17)  (n = 104) | 0.98 (0.89, 1.07)  (n = 161) |

^a^To assess the possibility of reverse causation, individuals with less than two years of follow-up were excluded from this analysis, regardless of whether they were a case of cancer or lost to follow-up within two years. Multivariable models were adjusted for age, sex, BMI, self-reported smoking status, self-reported smoking pack-years, postmenopausal hormone use (creating an ‘NA’ category for males), methylation-derived smoking pack-years, mdNLR (in all models except mdNLR and Lymphocyte to Monocyte ratio), and batch effect.

^b^ Excluding hematologic cancers. ^c^ The breast cancer model also adjusted for self-reported drinking status and did not adjust for sex. ^d^ The prostate cancer model did not adjust for sex or postmenopausal hormone use (men only).

Supplementary Table 5: HRs for Sex-Stratified Model in All Cancers ^a^:

HR ^b^ (95% CI) per 1 percent increase in methylation-derived immune cell proportion or 1 unit increase in ratios or white blood cell count

|  | Sex | |  |
| --- | --- | --- | --- |
| Methylation-Derived Immune Cell  Type or Measure | Females  (343 cases) | Males  (325 cases) | p-value for interaction |
| CD4+ | 0.99 (0.97, 1.00) | 1.00 (0.98, 1.02) | 0.25 |
| Memory CD4+ | 0.99 (0.97, 1.01) | 0.99 (0.97, 1.02) | 0.48 |
| Naïve CD4+ | 0.99 (0.97, 1.01) | 1.01 (0.98, 1.04) | 0.31 |
| Naïve-to-memory CD4+ ratio | 1.03 (0.99, 1.07) | 0.98 (0.92, 1.05) | 0.13 |
| CD8+ | 0.99 (0.97, 1.01) | 1.01 (0.99, 1.02) | 0.12 |
| Memory CD8+ | 1.00 (0.98, 1.02) | 1.01 (0.99, 1.02) | 0.19 |
| Naïve CD8+ | 0.96 (0.90, 1.02) | 0.95 (0.87, 1.04) | 0.63 |
| Naïve-to-memory CD8+ ratio | 0.98 (0.85, 1.14) | 0.97 (0.82, 1.16) | 0.61 |
| CD4+-to-CD8+ ratio | 0.98 (0.90, 1.07) | 0.97 (0.88, 1.05) | 0.71 |
| Treg | 1.06 (0.99, 1.14) | 1.03 (0.95, 1.13) | 0.08 |
| B cell | 1.01 (0.97, 1.04) | 0.97 (0.93, 1.01) | 0.22 |
| Memory B cell | 1.09 (0.97, 1.22) | **1.16 (1.05, 1.28)** | 0.22 |
| Naïve B cell | 1.00 (0.96, 1.03) | **0.95 (0.91, 0.99)** | 0.05 |
| Naïve-to-memory B cell ratio | 1.00 (0.98, 1.01) | 0.98 (0.97, 1.00) | 0.08 |
| NLR | 1.00 (0.97, 1.02) | 1.00 (0.97, 1.03) | 0.67 |
| Lymphocyte to Monocyte ratio | 1.00 (1.00, 1.00) | 1.02 (0.96, 1.10) | 0.29 |
| White blood cell count | **1.09 (1.02, 1.16)**  (n = 337) | 0.98 (0.92, 1.05)  (n = 322) | 0.01 |

^a^ Excluding hematologic cancers.

^b^ Multivariable models were adjusted for BMI, age, self-reported smoking status, self-reported smoking pack-years, methylation-derived smoking pack-years, mdNLR, post-menopausal hormone status (for women only), and batch effect.

Supplementary Table 6: HRs for Sex-Stratified Model in Lung Cancer:

HR (95% CI) per 1 percent increase in methylation-derived immune cell proportion or 1 unit increase in ratios or white blood cell count

|  | Sex | |  |
| --- | --- | --- | --- |
| Methylation-Derived Immune Cell Type or Measure | Females  (37 cases) | Males  (47 cases) | p-value for  interaction |
| CD4+ | 0.99 (0.93, 1.04) | 1.01 (0.96, 1.06) | 0.22 |
| Memory CD4+ | 0.99 (0.93, 1.06) | 1.01 (0.95, 1.07) | 0.22 |
| Naïve CD4+ | 0.98 (0.91, 1.06) | 1.02 (0.94, 1.11) | 0.67 |
| Naïve-to-memory CD4+ ratio | 0.90 (0.75, 1.08) | 1.01 (0.82, 1.24) | 0.85 |
| CD8+ | 0.99 (0.93, 1.06) | 1.04 (1.00, 1.08) | 0.16 |
| Memory CD8+ | 1.01 (0.95, 1.07) | 1.04 (1.00, 1.08) | 0.21 |
| Naïve CD8+ | 0.88 (0.71, 1.09) | 0.77 (0.57, 1.06) | 0.26 |
| Naïve-to-memory CD8+ ratio | 0.84 (0.49, 1.42) | 0.91 (0.52, 1.61) | 0.74 |
| CD4+-to-CD8+ ratio | 0.99 (0.77, 1.26) | 0.99 (0.81, 1.21) | 0.99 |
| Treg | 1.13 (0.93, 1.37) | 1.16 (0.92, 1.45) | 0.10 |
| B cell | 0.93 (0.82, 1.05) | 1.02 (0.91, 1.13) | 0.92 |
| Memory B cell | 1.10 (0.78, 1.53) | **1.34 (1.04, 1.71)** | 0.19 |
| Naïve B cell | 0.92 (0.81, 1.05) | 0.97 (0.84, 1.09) | 0.59 |
| Naïve-to-memory B cell ratio | 0.95 (0.91, 1.00) | 0.97 (0.93, 1.02) | 0.50 |
| NLR | 0.91 (0.77, 1.07) | 0.98 (0.89, 1.09) | 0.76 |
| Lymphocyte to Monocyte ratio | 1.00 (1.00, 1.00) | 1.08 (0.91, 1.29) | 0.19 |
| White blood cell count | **1.31 (1.09, 1.58)**  (n = 36) | 1.09 (0.94, 1.26)  (n = 47) | 0.12 |

Multivariable models were adjusted for BMI, age, self-reported smoking status, self-reported smoking pack-years, methylation-derived smoking pack-years, mdNLR, post-menopausal hormone status (for women only), and batch effect.

Supplementary Table 7: Immune Cell Measurement Percentiles:

|  | Percentile | | | | |  |
| --- | --- | --- | --- | --- | --- | --- |
| Methylation-Derived Immune Cell  Type or Measure | 10% | 25% | 50% | 75% | 90% | 90% to 10% Difference |
| CD4+ (%) | 3.67 | 8.06 | 13.13 | 18.48 | 24.35 | 20.68 |
| Memory CD4+ (%) | 1.06 | 2.97 | 7.32 | 12.01 | 16.26 | 15.20 |
| Naïve CD4+ (%) | 0.86 | 2.16 | 4.97 | 8.28 | 11.95 | 11.09 |
| Naïve-to-memory CD4+ ratio | 0.11 | 0.32 | 0.78 | 1.64 | 4.05 | 3.94 |
| CD8+ (%) | 5.00 | 8.25 | 12.14 | 16.45 | 21.44 | 16.44 |
| Memory CD8+ (%) | 3.14 | 6.10 | 10.10 | 15.06 | 20.51 | 17.37 |
| Naïve CD8+ (%) | 0.79 | 0.79 | 1.04 | 2.66 | 4.25 | 3.46 |
| Naïve-to-memory CD8+ ratio | 0.04 | 0.06 | 0.15 | 0.39 | 0.99 | 0.95 |
| CD4+-to-CD8+ ratio | 0.36 | 0.65 | 1.10 | 1.72 | 2.59 | 2.23 |
| Treg (%) | 0.80 | 0.80 | 1.57 | 2.83 | 4.19 | 3.39 |
| B cell (%) | 1.62 | 2.97 | 4.80 | 7.05 | 9.48 | 7.86 |
| Memory B cell (%) | 0.38 | 0.38 | 0.38 | 0.38 | 1.41 | 1.03 |
| Naïve B cell (%) | 1.31 | 2.63 | 4.41 | 6.52 | 8.98 | 7.67 |
| Naïve-to-memory B cell ratio | 1.46 | 4.35 | 9.76 | 15.39 | 22.50 | 21.04 |
| NLR | 0.63 | 0.87 | 1.32 | 2.09 | 3.65 | 3.02 |
| Lymphocyte to Monocyte ratio | 1.97 | 2.70 | 3.57 | 4.62 | 5.78 | 3.81 |
| White blood cell count (x1000/mm^3^) | 3.70 | 4.40 | 5.30 | 6.50 | 8.00 | 4.30 |

Supplementary Table 8: Immune Cell Measurement Boundaries for Tertiles:

|  | Tertile | |
| --- | --- | --- |
| Methylation-Derived Immune Cell  Type or Measure | Tertile 1-2  Cutoff | Tertile 2-3  Cutoff |
| CD4+ (%) | 9.89 | 16.75 |
| Memory CD4+ (%) | 4.70 | 10.40 |
| Naïve CD4+ (%) | 3.06 | 6.98 |
| Naïve-to-memory CD4+ ratio | 0.45 | 1.20 |
| CD8+ (%) | 9.57 | 14.88 |
| Memory CD8+ (%) | 7.39 | 13.27 |
| Naïve CD8+ (%) | 0.79 | 2.08 |
| Naïve-to-memory CD8+ ratio | 0.08 | 0.28 |
| CD4+-to-CD8+ ratio | 0.80 | 1.45 |
| Treg (%) | 0.80 | 2.38 |
| B cell (%) | 3.65 | 6.04 |
| Memory B cell (%) | 0.38 | 0.38 |
| Naïve B cell (%) | 3.30 | 5.63 |
| Naïve-to-memory B cell ratio | 6.11 | 13.40 |
| NLR | 0.99 | 1.79 |
| Lymphocyte to Monocyte ratio | 2.99 | 4.22 |
| White blood cell count (x1000/mm^3^) | 4.70 | 6.10 |

Supplementary Table 9: HRs for Quartile Analysis in All Cancers :

HR ^a^ (95% CI) for each quartile of methylation-derived immune cell proportion, ratios, or white blood cell count

|  | Quartile | | | |  | |
| --- | --- | --- | --- | --- | --- | --- |
| Methylation-Derived Immune Cell  Type or Measure | Quartile 1 HR  (95% CI) | Quartile 2 HR  (95% CI) | Quartile 3 HR  (95% CI) | Quartile 4 HR  (95% CI) | | p-trend |
| CD4+ | Ref.  (n = 182) | 0.88 (0.70, 1.10)  (n = 165) | 0.89 (0.71, 1.12)  (n = 168) | 0.89 (0.70, 1.13)  (n = 153) | | 0.33 |
| Memory CD4+ | Ref.  (n = 175) | 0.93 (0.74, 1.16)  (n = 161) | 0.90 (0.71, 1.13)  (n = 173) | 0.87 (0.68, 1.11)  (n = 159) | | 0.26 |
| Naïve CD4+ | Ref.  (n = 181) | 0.92 (0.74, 1.14)  (n = 166) | 0.96 (0.77, 1.20)  (n = 167) | 0.98 (0.78, 1.24)  (n = 154) | | 0.99 |
| Naïve-to-memory CD4+ ratio | Ref.  (n = 168) | 1.15 (0.93, 1.42)  (n = 173) | 0.96 (0.76, 1.21)  (n = 152) | 1.21 (0.96, 1.52)  (n = 175) | | 0.30 |
| CD8+ | Ref.  (n = 180) | 1.01 (0.81, 1.26)  (n = 161) | 0.92 (0.73, 1.15)  (n = 154) | 1.05 (0.83, 1.31)  (n = 173) | | 0.83 |
| Memory CD8+ | Ref.  (n = 178) | 0.97 (0.78, 1.21)  (n = 166) | 0.83 (0.66, 1.05)  (n = 146) | 1.05 (0.84, 1.31)  (n = 178) | | 0.49 |
| Naïve CD8+ | Ref.  (n = 322) | 1.02 (0.83, 1.25)  (n = 134) | 0.80 (0.64, 1.01)  (n = 105) | 0.83 (0.66, 1.04)  (n = 107) | | 0.09 |
| Naïve-to-memory CD8+ ratio | Ref.  (n = 182) | 0.95 (0.77, 1.17)  (n = 172) | 0.83 (0.67, 1.03)  (n = 153) | 0.87 (0.69, 1.09)  (n = 161) | | 0.67 |
| CD4+-to-CD8+ ratio | Ref.  (n = 196) | **0.74 (0.60, 0.91)**  (n = 150) | **0.76 (0.61, 0.94)**  (n = 154) | 0.85 (0.69, 1.06)  (n = 168) | | 0.39 |
| Treg | Ref.  (n = 201) | **1.30 (1.03, 1.64)**  (n = 114) | 1.24 (1.00, 1.53)  (n = 166) | **1.31 (1.04, 1.65)**  (n = 187) | | 0.05 |
| B cell | Ref.  (n = 177) | 0.83 (0.66, 1.04)  (n = 156) | 0.89 (0.71, 1.11)  (n = 170) | 0.93 (0.74, 1.18)  (n = 165) | | 0.61 |
| B cell memory | Ref.  (n = 496) | 0.73 (0.53, 1.01)  (n = 40) | 1.16 (0.88, 1.53)  (n = 60) | **1.47 (1.13, 1.91)**  (n = 72) | | 0.001 |
| Naïve B cell | Ref.  (n = 187) | **0.79 (0.63, 0.98)**  (n = 165) | **0.77 (0.61, 0.96)**  (n = 161) | **0.79 (0.62, 0.99)**  (n = 155) | | 0.14 |
| Naïve-to-memory B cell ratio | Ref.  (n = 186) | 0.91 (0.74, 1.12)  (n = 181) | **0.76 (0.61, 0.96)**  (n = 150) | 0.81 (0.64, 1.02)  (n = 151) | | 0.08 |
| NLR | Ref.  (n = 154) | 1.00 (0.80, 1.25)  (n = 159) | 1.08 (0.87, 1.34)  (n = 177) | 1.14 (0.91, 1.42)  (n = 178) | | 0.96 |
| Lymphocyte to Monocyte ratio | Ref.  (n = 177) | 0.90 (0.73, 1.12)  (n = 161) | 1.09 (0.88, 1.35)  (n = 174) | 1.01 (0.80, 1.26)  (n = 156) | | 0.81 |
| White blood cell count | Ref.  (n = 165) | 1.04 (0.83, 1.31)  (n = 149) | 1.16 (0.94, 1.45)  (n = 174) | 1.15 (0.92, 1.44)  (n = 171) | | 0.21 |

^a^ Excluding hematologic cancers. Multivariable models were adjusted for age, sex, BMI, self-reported smoking status, self-reported smoking pack-years, methylation-derived smoking pack-years, postmenopausal hormone use, mdNLR, and batch effect.

Supplementary Table 10: HRs for Tertile Analysis in Lung Cancer:

HR (95% CI) for each tertile of methylation-derived immune cell proportion, ratios, or white blood cell count

|  | Tertile | | |  |
| --- | --- | --- | --- | --- |
| Methylation-Derived Immune Cell  Type or Measure | Tertile 1 HR  (95% CI) | Tertile 2 HR  (95% CI) | Tertile 3 HR  (95% CI) | p-trend |
| CD4+ | Ref.  (n = 22) | 1.60 (0.90, 2.85)  (n = 38) | 1.10 (0.57, 2.14)  (n = 24) | 0.81 |
| Memory CD4+ | Ref.  (n = 22) | 1.42 (0.80, 2.52)  (n = 38) | 0.83 (0.42, 1.63)  (n = 24) | 0.79 |
| Naïve CD4+ | Ref.  (n = 34) | 0.70 (0.41, 1.20)  (n = 24) | 0.97 (0.56, 1.69)  (n = 26) | 0.98 |
| Naïve-to-memory CD4+ ratio | Ref.  (n = 35) | 0.76 (0.44, 1.31)  (n = 24) | 1.10 (0.62, 1.93)  (n = 25) | 0.56 |
| CD8+ | Ref.  (n = 31) | 0.84 (0.48, 1.47)  (n = 25) | 1.03 (0.59, 1.79)  (n = 28) | 0.16 |
| Memory CD8+ | Ref.  (n = 26) | 1.28 (0.74, 2.22)  (n = 31) | 1.27 (0.71, 2.27)  (n = 27) | 0.06 |
| Naïve CD8+ | Ref.  (n = 46) | 0.67 (0.40, 1.13)  (n = 23) | **0.47 (0.25, 0.87)**  (n = 15) | 0.06 |
| Naïve-to-memory CD8+ ratio | Ref.  (n = 34) | 0.87 (0.52, 1.46)  (n = 28) | 0.57 (0.32, 1.01)  (n = 22) | 0.49 |
| CD4+-to-CD8+ ratio | Ref.  (n = 35) | **0.54 (0.31, 0.94)**  (n = 20) | 0.73 (0.44, 1.22)  (n = 29) | 0.82 |
| Treg | Ref.  (n = 20) | 1.26 (0.69, 2.28)  (n = 26) | 1.42 (0.77, 2.63)  (n = 38) | 0.01 |
| B cell | Ref.  (n = 27) | 0.83 (0.47, 1.46)  (n = 27) | 0.99 (0.56, 1.77)  (n = 30) | 0.66 |
| Memory B cell | Ref.  (n = 54) | 1.27 (0.68, 2.36)  (n = 14) | 1.56 (0.84, 2.89)  (n = 16) | 0.60 |
| Naïve B cell | Ref.  (n = 30) | 0.81 (0.47, 1.39)  (n = 29) | 0.74 (0.41, 1.32)  (n = 25) | 0.29 |
| Naïve-to-memory B cell ratio | Ref.  (n = 37) | 0.77 (0.46, 1.30)  (n = 27) | **0.53 (0.29, 0.97)**  (n = 20) | 0.06 |
| NLR | Ref.  (n = 19) | **1.78 (1.01, 3.14)**  (n = 35) | 1.17 (0.64, 2.12)  (n = 30) | 0.21 |
| Lymphocyte to Monocyte ratio | Ref.  (n = 26) | 1.53 (0.91, 2.57)  (n = 34) | 1.29 (0.72, 2.33)  (n = 24) | 0.99 |
| White blood cell count | Ref.  (n = 15) | 1.73 (0.91, 3.27)  (n = 28) | **2.67 (1.43, 4.97)**  (n = 40) | 0.002 |

Multivariable models were adjusted for age, sex, BMI, self-reported smoking status, self-reported smoking pack-years, methylation-derived smoking pack-years, postmenopausal hormone use, mdNLR, and batch effect.

Supplementary Table 11: HRs for Tertile Analysis in Postmenopausal Breast Cancer:

HR (95% CI) for each tertile of methylation-derived immune cell proportion, ratio, or white blood cell count

|  | Tertile | | |  |
| --- | --- | --- | --- | --- |
| Methylation-Derived Immune Cell  Type or Measure | Tertile 1 HR  (95% CI) | Tertile 2 HR  (95% CI) | Tertile 3 HR  (95% CI) | p-trend |
| CD4+ | Ref.  (n = 33) | 0.97 (0.59, 1.58)  (n = 40) | 0.85 (0.51, 1.41)  (n = 41) | 0.78 |
| Memory CD4+ | Ref.  (n = 37) | 0.84 (0.50, 1.39)  (n = 32) | 1.08 (0.65, 1.79)  (n = 45) | 0.41 |
| Naïve CD4+ | Ref.  (n = 33) | 1.02 (0.64, 1.63)  (n = 44) | 0.72 (0.44, 1.20)  (n = 37) | 0.18 |
| Naïve-to-memory CD4+ ratio | Ref.  (n = 41) | 0.71 (0.45, 1.15)  (n = 34) | 0.76 (0.47, 1.23)  (n = 39) | 0.95 |
| CD8+ | Ref.  (n = 41) | 0.66 (0.40, 1.07)  (n = 30) | 0.93 (0.59, 1.47)  (n = 43) | 0.34 |
| Memory CD8+ | Ref.  (n = 42) | **0.61 (0.37, 0.99)**  (n = 27) | 1.10 (0.71, 1.72)  (n = 45) | 0.21 |
| Naïve CD8+ | Ref.  (n = 62) | **0.36 (0.20, 0.63)**  (n = 16) | **0.64 (0.41, 0.99)**  (n = 36) | 0.47 |
| Naïve-to-memory CD8+ ratio | Ref.  (n = 45) | **0.60 (0.37, 0.96)**  (n = 28) | 0.73 (0.46, 1.15)  (n = 41) | 0.76 |
| CD4+-to-CD8+ ratio | Ref.  (n = 34) | 1.09 (0.70, 1.72)  (n = 46) | 0.83 (0.51, 1.36)  (n = 34) | 0.23 |
| Treg | Ref.  (n = 34) | 0.75 (0.44, 1.28)  (n = 25) | 1.49 (0.92, 2.43)  (n = 55) | 0.51 |
| B cell | Ref.  (n = 29) | 0.85 (0.51, 1.43)  (n = 34) | 1.14 (0.70, 1.86)  (n = 51) | 0.22 |
| Memory B cell | Ref.  (n = 94) | 0.44 (0.19, 1.01)  (n = 6) | 1.20 (0.65, 2.19)  (n = 14) | 0.72 |
| Naïve B cell | Ref.  (n = 25) | 1.05 (0.61, 1.78)  (n = 36) | 1.40 (0.84, 2.34)  (n = 53) | 0.17 |
| Naïve-to-memory B cell ratio | Ref.  (n = 30) | 0.82 (0.49, 1.36)  (n = 33) | 1.12 (0.68, 1.84)  (n = 51) | 0.18 |
| NLR | Ref.  (n = 42) | 0.97 (0.62, 1.51)  (n = 38) | 1.02 (0.65, 1.61)  (n = 34) | 0.70 |
| Lymphocyte to Monocyte ratio | Ref.  (n = 27) | 0.96 (0.57, 1.61)  (n = 33) | 1.31 (0.81, 2.09)  (n = 54) | 0.84 |
| White blood cell count | Ref.  (n = 36) | 1.10 (0.70, 1.75)  (n = 40) | 1.22 (0.75, 1.98)  (n = 36) | 0.37 |

Multivariable models were adjusted for BMI, self-reported smoking status, self-reported smoking pack-years, methylation-derived smoking pack-years, self-reported drinking status, postmenopausal hormone use, mdNLR, and batch effect.

Supplementary Table 12: HRs for Tertile Analysis in Prostate Cancer:

HR (95% CI) for each tertile of methylation-derived immune cell proportion, ratio,

or white blood cell count

|  | Tertile | | |  |
| --- | --- | --- | --- | --- |
| Methylation-Derived Immune Cell  Type or Measure | Tertile 1 HR  (95% CI) | Tertile 2 HR  (95% CI) | Tertile 3 HR  (95% CI) | p-trend |
| CD4+ | Ref.  (n = 68) | 1.08 (0.74, 1.57)  (n = 59) | 1.10 (0.74, 1.65)  (n = 46) | 0.69 |
| Memory CD4+ | Ref.  (n = 59) | 1.19 (0.81, 1.75)  (n = 62) | 1.13 (0.74, 1.72)  (n = 52) | 0.95 |
| Naïve CD4+ | Ref.  (n = 75) | 0.85 (0.59, 1.22)  (n = 53) | 1.02 (0.70, 1.51)  (n = 45) | 0.56 |
| Naïve-to-memory CD4+ ratio | Ref.  (n = 64) | 1.13 (0.79, 1.64)  (n = 60) | 0.95 (0.64, 1.41)  (n = 49) | 0.61 |
| CD8+ | Ref.  (n = 56) | 1.25 (0.84, 1.87)  (n = 53) | 1.40 (0.95, 2.06)  (n = 64) | 0.31 |
| Memory CD8+ | Ref.  (n = 57) | 1.06 (0.71, 1.59)  (n = 49) | 1.24 (0.85, 1.82)  (n = 67) | 0.38 |
| Naïve CD8+ | Ref.  (n = 84) | **1.47 (1.05, 2.07)**  (n = 60) | 0.89 (0.57, 1.38)  (n = 29) | 0.98 |
| Naïve-to-memory CD8+ ratio | Ref.  (n = 63) | 1.21 (0.86, 1.72)  (n = 67) | 0.79 (0.52, 1.20)  (n = 43) | 0.24 |
| CD4+-to-CD8+ ratio | Ref.  (n = 71) | 0.87 (0.60, 1.24)  (n = 53) | 0.84 (0.58, 1.22)  (n = 49) | 0.35 |
| Treg | Ref.  (n = 55) | **1.59 (1.10, 2.31)**  (n = 66) | 1.34 (0.87, 2.06)  (n = 52) | 0.20 |
| B cell | Ref.  (n = 81) | 0.83 (0.57, 1.20)  (n = 50) | 0.87 (0.59, 1.29)  (n = 42) | 0.32 |
| Memory B cell | Ref.  (n = 127) | 0.76 (0.45, 1.30)  (n = 16) | 1.27 (0.83, 1.95)  (n = 30) | 0.01 |
| Naïve B cell | Ref.  (n = 85) | 0.77 (0.54, 1.11)  (n = 51) | 0.70 (0.47, 1.06)  (n = 37) | 0.06 |
| Naïve-to-memory B cell ratio | Ref.  (n = 85) | **0.68 (0.47, 0.98)**  (n = 50) | 0.72 (0.48, 1.08)  (n = 38) | 0.10 |
| NLR | Ref.  (n = 55) | 0.96 (0.66, 1.40)  (n = 56) | 0.91 (0.63, 1.31)  (n = 62) | 0.59 |
| Lymphocyte to Monocyte ratio | Ref.  (n = 71) | 1.12 (0.79, 1.59)  (n 60) | 1.16 (0.79, 1.71)  (n = 42) | 0.47 |
| White blood cell count | Ref.  (n = 66) | 1.12 (0.78, 1.63)  (n = 56) | 0.95 (0.64, 1.40)  (n = 49) | 0.80 |

Multivariable models were adjusted for BMI, self-reported smoking status, self-reported smoking pack-years, methylation-derived smoking pack-years, mdNLR, and batch effect.

Supplementary 13: HRs for Individuals with White Blood Cell Count in Normal Range^a^:

HR (95% CI) per 1 percent increase in methylation-derived immune cell proportion or 1 unit increase in ratios or white blood cell count

| Methylation-Derived Immune Cell Type or Measure | All Cancer  (630 cases) | Lung Cancer  (78 cases) | Postmenopausal Breast Cancer ^b^  (107 cases) | Prostate Cancer ^c^  (165 cases) |
| --- | --- | --- | --- | --- |
| CD4+ | 1.00 (0.92, 1.03) | 1.00 (0.96, 1.04) | 0.99 (0.97, 1.02) | 1.01 (0.98, 1.03) |
| Memory CD4+ | 1.00 (0.97, 1.03) | 1.00 (0.95, 1.04) | 1.01 (0.97, 1.04) | 1.00 (0.97, 1.03) |
| Naïve CD4+ | 1.00 (0.95, 1.04) | 1.00 (0.94, 1.06) | 0.97 (0.93, 1.02) | 1.02 (0.98, 1.07) |
| Naïve-to-memory CD4+ ratio | 0.97 (0.89, 1.07) | 0.96 (0.83, 1.10) | 1.01 (0.94, 1.09) | 0.99 (0.91, 1.07) |
| CD8+ | 1.02 (0.99, 1.04) | 1.03 (0.99, 1.06) | 1.02 (0.99, 1.05) | 1.01 (0.99, 1.03) |
| Memory CD8+ | 1.01 (0.99, 1.04) | **1.03 (1.01, 1.07)** | 1.02 (0.99, 1.05) | 1.01 (0.99, 1.03) |
| Naïve CD8+ | 0.99 (0.87, 1.11) | **0.81 (0.67, 0.99)** | 0.97 (0.84, 1.14) | 1.04 (0.92, 1.17) |
| Naïve-to-memory CD8+ ratio | 0.75 (0.52, 1.09) | 0.88 (0.58, 1.32) | 0.97 (0.75, 1.27) | 0.82 (0.59, 1.14) |
| CD4+-to-CD8+ ratio | 0.93 (0.80, 1.08) | 0.99 (0.85, 1.15) | 0.97 (0.73, 1.06) | 0.93 (0.82, 1.07) |
| Treg | 1.06 (0.93, 1.21) | **1.19 (1.02, 1.38)** | 1.06 (0.94, 1.21) | 1.10 (0.97, 1.24) |
| B cell | 0.97 (0.91, 1.03) | 0.99 (0.91, 1.08) | 1.04 (0.97, 1.10) | 0.97 (0.92, 1.03) |
| Memory B cell | **1.19 (1.05, 1.34)** | **1.21 (1.01, 1.47)** | 0.93 (0.70, 1.23) | **1.15 (1.02, 1.31)** |
| Naïve B cell | 0.94 (0.88, 1.00) | 0.96 (0.88, 1.05) | 1.04 (0.98, 1.11) | 0.95 (0.89, 1.01) |
| Naïve-to-memory B cell ratio | 0.98 (0.96, 1.00) | 0.97 (0.94, 1.00) | 1.02 (0.99, 1.04) | 0.98 (0.96, 1.01) |
| NLR | 0.99 (0.91, 1.08) | 0.80 (0.57, 1.13) | 0.99 (0.95, 1.04) | 0.99 (0.95, 1.03) |
| Lymphocyte to Monocyte ratio | 0.92 (0.47, 1.80) | 1.00 (1.00, 1.00) | 1.00 (1.00, 1.00) | 1.05 (0.97, 1.13) |
| White blood cell count | 1.02 (0.97, 1.08)  (n = 630) | 1.15 (1.00, 1.33)  (n = 78) | 1.07 (0.94, 1.23)  (n = 107) | 0.96 (0.86, 1.07)  (n = 165) |

^a^In order to analyze cancer risk in participants who fall within reference white blood cell count levels, this analysis was conducted excluding all African American participants who fell outside of the white blood cell count reference range for African Americans (women: 3.4-11.0 × 10^9^/l, men: 3.1-9.9 × 10^9^/l). ^b^ The breast cancer model also adjusted for self-reported drinking status and did not adjust for sex. ^c^ The prostate cancer model did not adjust for sex or postmenopausal hormone use (men only).

Supplementary Figure 1: Postmenopausal Breast Cancer Spline Plots:


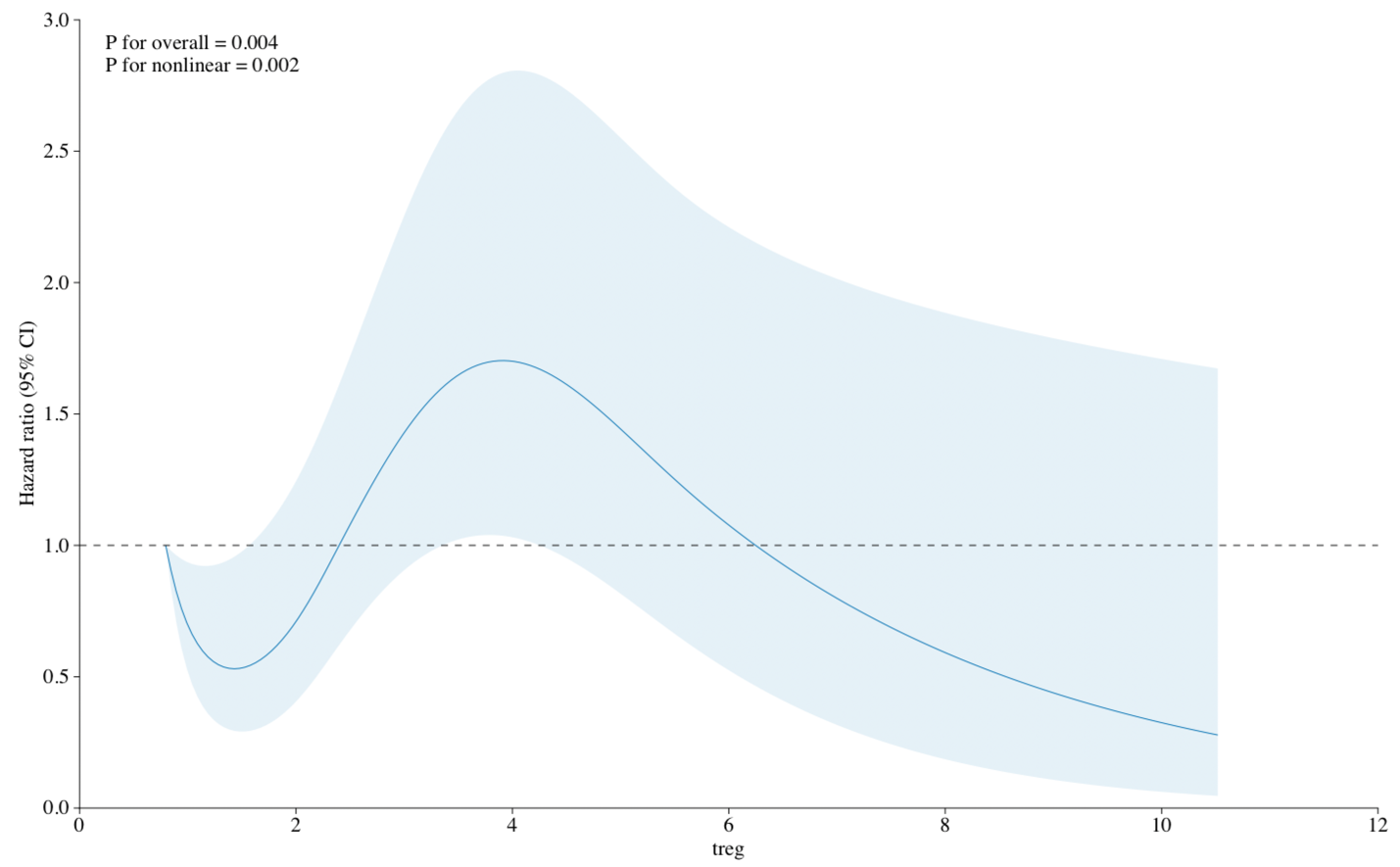


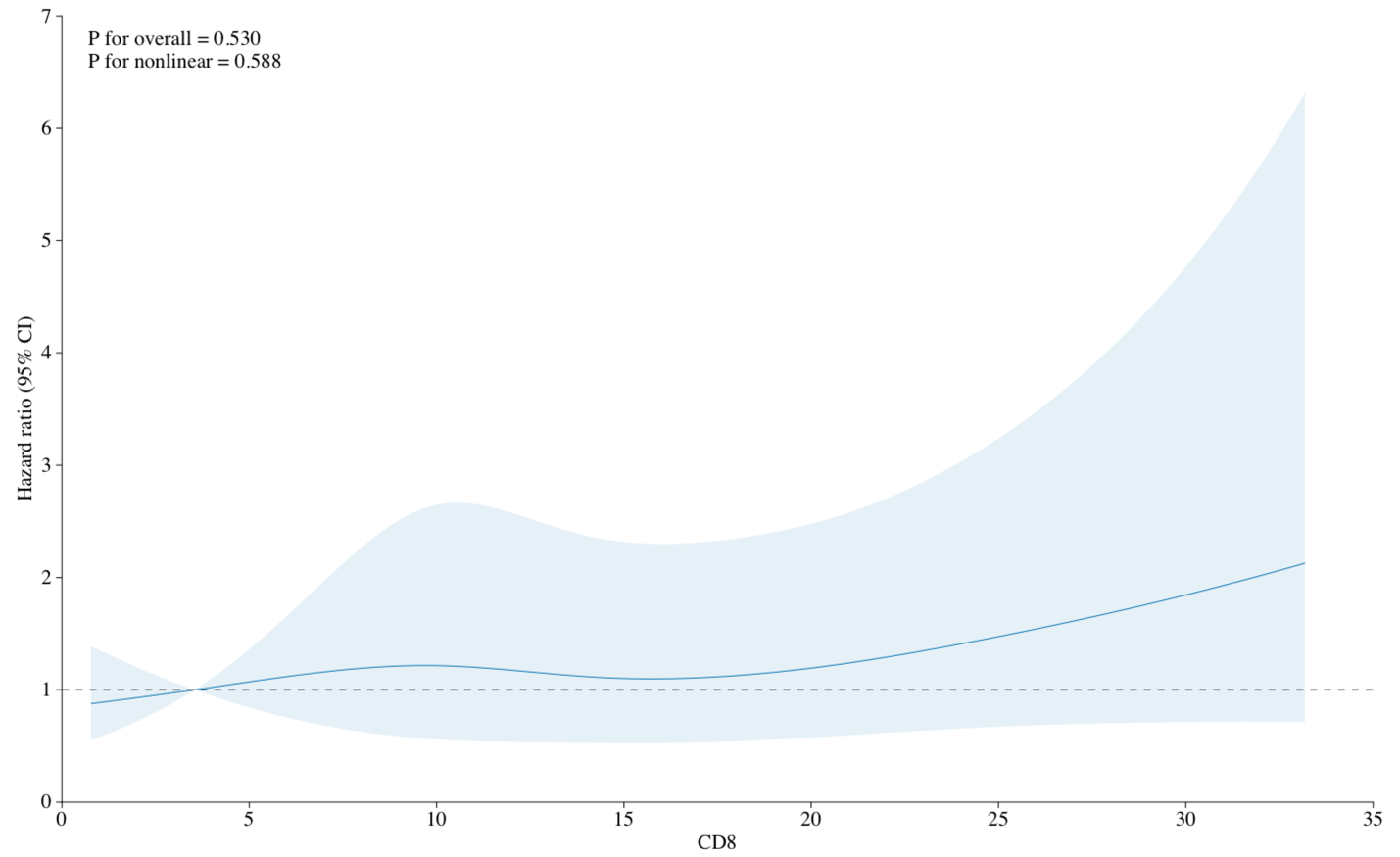


These spline plots assess the changes in the hazard ratios at various proportions of Tregs and CD8+ cells in breast cancer. The P for nonlinear for Tregs of 0.002 and P for nonlinear of 0.588 for CD8+ cells call into question the appropriateness of a linear model for assessing this relationship.

Supplementary Figure 2: Prostate Cancer Spline Plots:


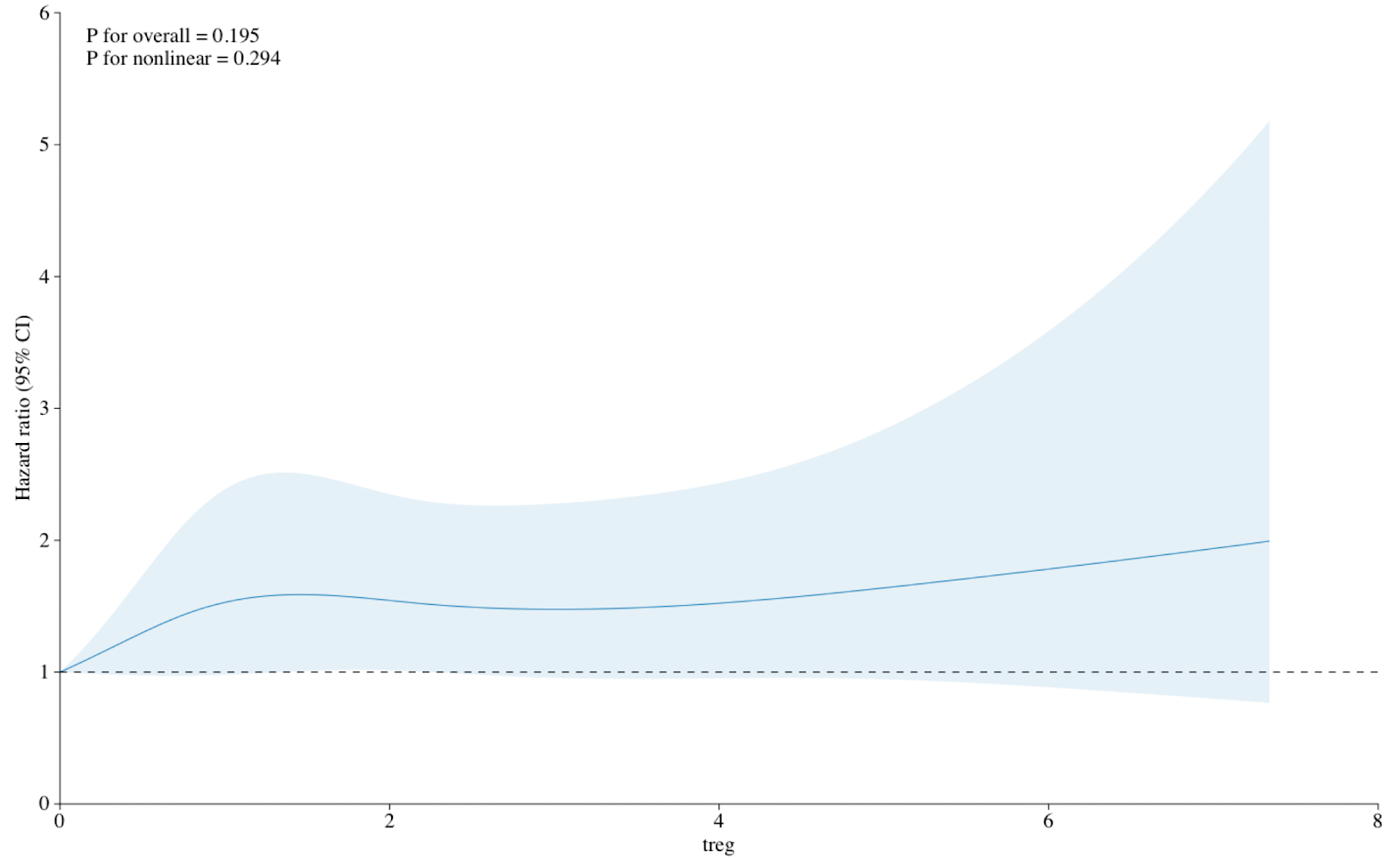


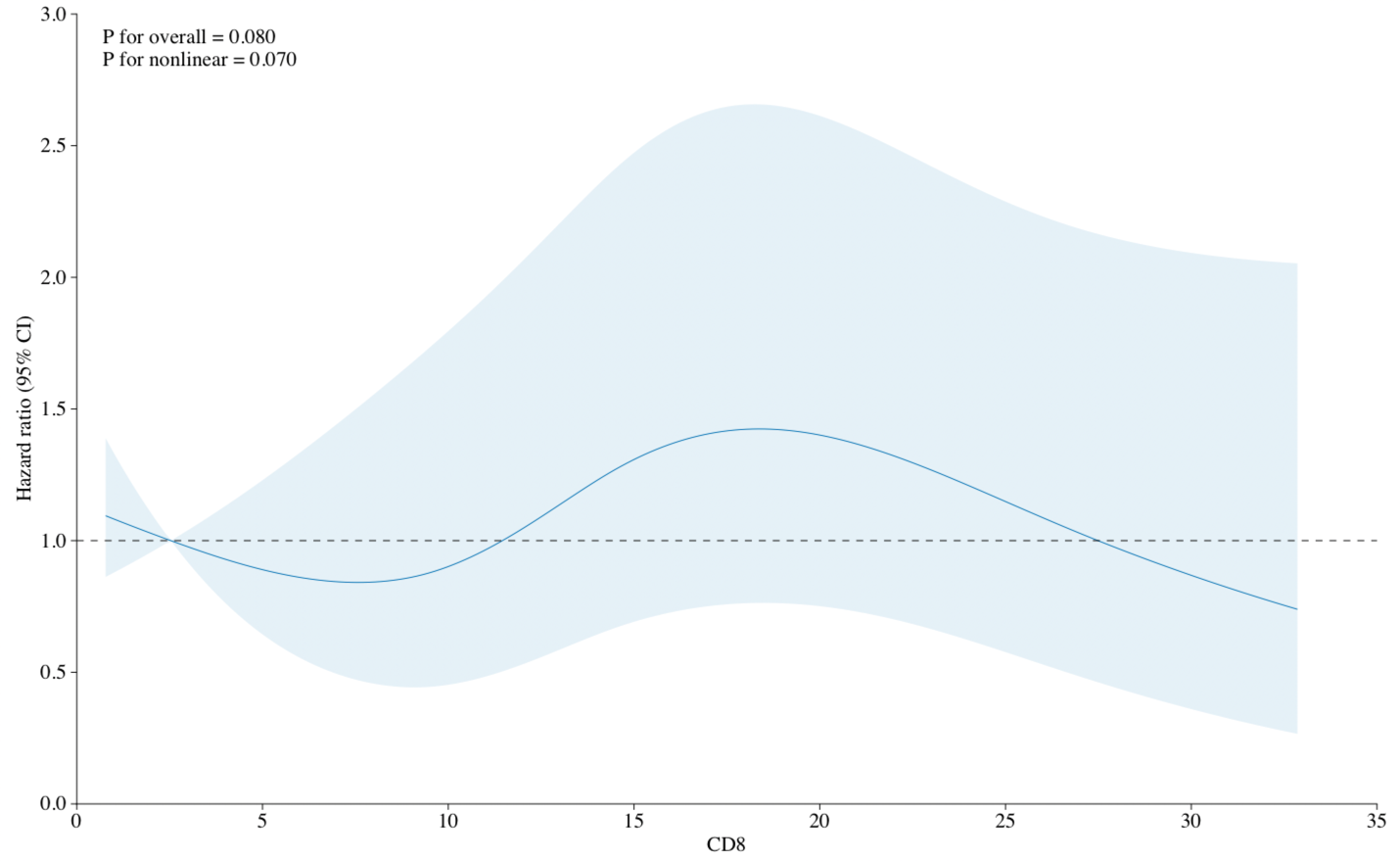


These spline plots assess the changes in the hazard ratios at various proportions of Tregs and CD8+ cells in prostate cancer. The P for nonlinear for Tregs of 0.294 and P for nonlinear of 0.070 for CD8+ cells indicates that a linear model is likely an appropriate means of representing these associations.
